# Access to adults’ hearing aids: policies and technologies used in eight countries

**DOI:** 10.2471/BLT.18.228676

**Published:** 2019-08-20

**Authors:** Michael Yong, Amber Willink, Catherine McMahon, Bradley McPherson, Carrie L Nieman, Nicholas S Reed, Frank R Lin

**Affiliations:** a Cochlear Center for Hearing and Public Health, Johns Hopkins University Bloomberg School of Public Health, 2024 E Monument St. Suite 2-700 Baltimore, Maryland, United States of America.; b Macquarie University Research Centre H:EAR, Sydney, Australia.; c Faculty of Education, University of Hong Kong, Hong Kong, China.

## Abstract

As the proportion of older adults in the world’s total population continues to grow, the adverse health outcomes of age-related hearing loss are becoming increasingly recognized. While research has shown that age-related hearing loss is the single greatest modifiable risk factor for dementia, use of hearing aids remains low worldwide, even in many middle- and high-income countries. Reasons for poor uptake of hearing aids are likely to involve a combination of factors, ranging from increasing costs of hearing aid technology to a widespread lack of insurance coverage. This article aims to identify the current state of access to hearing aids, focusing on eight middle- and high-income countries. We discuss how to facilitate greater access to hearing aids for patients by addressing changes in how devices are regulated, technological advancements in hearing devices, the need to adjust reimbursement schemes and the importance of adaptation among the community workforce for hearing-care.

## Résumé

Alors que la proportion de personnes âgées au sein de la population mondiale totale continue à croître, les effets néfastes sur la santé de la perte de l'acuité auditive liée à l'âge sont de plus en plus reconnus. Bien que la recherche ait démontré que la perte de l'acuité auditive liée à l'âge est le principal facteur de risque modifiable de la démence, l'utilisation de prothèses auditives reste limitée à l'échelle mondiale, y compris dans de nombreux pays à revenu intermédiaire et élevé. Les raisons de ce recours limité aux prothèses auditives tiennent probablement à une combinaison de facteurs qui vont des coûts croissants de la technologie des appareils auditifs à un manque généralisé de couverture médicale. Cet article vise à déterminer l'état actuel de l'accès aux prothèses auditives en se concentrant sur huit pays à revenu intermédiaire et élevé. Nous étudions comment permettre aux patients d'accéder plus facilement aux prothèses auditives en tenant compte de la réglementation applicable aux appareils, des progrès technologiques relatifs aux appareils auditifs, de la nécessité d'ajuster les systèmes de remboursement et de l'importance de l'adaptation au sein de la main-d'œuvre locale pour les soins auditifs.

## Resumen

A medida que la proporción de adultos mayores en la población total del mundo continúa creciendo, los resultados adversos para la salud de la pérdida de audición relacionada con la edad son cada vez más reconocidos. Aunque las investigaciones han demostrado que la pérdida de audición relacionada con la edad es el mayor factor de riesgo modificable para la demencia, el uso de audífonos sigue siendo bajo en todo el mundo, incluso en muchos países de ingresos medios y altos. Las causas de la escasa aceptación de los audífonos pueden ser una combinación de factores, que van desde el aumento de los costes de la tecnología de los audífonos hasta la falta generalizada de cobertura de seguro. Este artículo pretende identificar el estado actual del acceso a los audífonos, centrándose en ocho países de ingresos medios y altos. Discutimos cómo facilitar un mayor acceso a los audífonos para los pacientes abordando los cambios en cómo se regulan los dispositivos, los avances tecnológicos en los audífonos, la necesidad de ajustar los esquemas de reembolso y la importancia de la adaptación entre los trabajadores de la comunidad para el cuidado de la audición.

## ملخص

مع استمرار الزيادة في نسبة المسنين من إجمالي عدد سكان العالم، فإن النتائج الصحية الضارة لفقدان السمع المرتبط بالتقدم في السن، أصبح من الممكن تمييزها بشكل متزايد. بينما أظهرت الأبحاث أن فقدان السمع المرتبط بالتقدم في السن هو أكبر عامل خطر والوحيد القابل للتعديل ومؤدي للخرف، إلا أن استخدام أدوات المساعدة السمعية لا زال منخفضاً في جميع أنحاء العالم، حتى في العديد من البلدان ذات الدخل المتوسط ​​والدخل المرتفع. من المحتمل أن تتضمن أسباب ضعف الإقبال على أدوات المساعدة السمعية العديد من العوامل، تتراوح من زيادة تكاليف تكنولوجيا أدوات المساعدة السمعية، إلى نقص واسع الانتشار في التغطية التأمينية. تهدف هذه المقالة إلى تعريف الوضع الحالي لإمكانية الحصول على أدوات المساعدة السمعية، والتركيز على ثمانية بلدان متوسطة الدخل ومرتفعة الدخل. نحن نناقش كيفية تسهيل حصول المرضى بشكل أكبر على أدوات المساعدة السمعية، وذلك عن طريق التركيز على التغييرات في طريقة تنظيم الأجهزة، والتطورات التكنولوجية في أجهزة المساعدة السمعية، والحاجة إلى ضبط نظم استرداد التكاليف، وأهمية التكيف بين القوى العاملة المجتمعية تجاه رعاية السمع.

## 摘要

随着老年人在世界人口总量中所占比例持续增长，与年龄相关的听力损失所带来的不利健康影响日益得到认同。研究表明，与年龄相关的听力损失是老年痴呆症最大的可变风险因素，但在全世界范围内，助听器的使用仍然很有限，即使在许多中高收入国家，现状也是如此。助听器使用率低有诸多原因，包括不断上升的技术成本以及设备不包含在保险覆盖范围内。本文旨在确定获取助听器的现状，着重关注八个中高收入国家。我们将讨论如何通过改变设备的监管方式、助听器的技术进步、调整补偿方案的必要性以及社区工作者适应听力保健的重要性，从而促进患者有更多的机会使用助听器。

## Резюме

Поскольку доля пациентов старшего возраста в общем населении земного шара продолжает расти, неблагоприятные для здоровья исходы возрастной потери слуха регистрируются все чаще. Несмотря на то что исследование показало, что возрастная потеря слуха является единственным наибольшим модифицируемым фактором риска деменции, использование слуховых аппаратов остается низким глобально, даже во многих странах со средним и высоким уровнем дохода. Низкий уровень применения слуховых аппаратов, вероятно, обусловлен сочетанием нескольких факторов: от увеличения стоимости технологий их производства до широкого распространения фактов отсутствия страхового покрытия. Целью данной статьи является определение текущего состояния доступа к слуховым аппаратам, проведенное в восьми странах со средним или высоким доходом. Обсуждаются возможности стимулирования более обширного доступа пациентов к слуховым аппаратам путем описания изменений законодательного контроля, технологических достижений, необходимости коррекции схем возмещения и важности адаптации среди сотрудников системы здравоохранения.

## Introduction

Policies around the world to address hearing loss have followed an advancing scientific understanding of the impact of hearing loss on health. Since the 1990s, worldwide efforts to screen for hearing loss in early childhood, followed by appropriate paediatric hearing interventions, have followed from evidence that hearing loss markedly impacts a child’s intellectual, social, and emotional development.^1^^,^^2^ Appropriately-timed interventions for childhood hearing, loss such as early use of amplification have been studied extensively and are now widely recognized as crucial to improving long-term health outcomes.^3^ However, more recently, in 2017, the World Health Assembly unanimously adopted a resolution for the prevention of deafness and hearing loss that calls on Member States to ensure that ear and hearing care is accessible to people across the lifespan in addition to early-life interventions.^4^

This focus on a life-course approach to hearing loss is timely given the projected global increase in the number and relative proportion of older adults over the next 30 years.^5^^,^^6^ While addressing hearing loss in children remains a global health priority, over 90% of the burden of hearing loss is experienced by older adults.^7^ Epidemiological estimates of hearing loss show that the prevalence of hearing loss nearly doubles with every increasing age decade, such that two thirds of all adults 70 years or older have a hearing loss that impairs daily communication.^8^^,^^9^ Furthermore, World Health Organization (WHO) estimates suggest that over two thirds of the burden of age-related hearing loss occurs in low- to middle-income countries and is greater in rural than urban areas, where resources to provide hearing health care are poor.^10^^,^^11^

Research over the past decade has now demonstrated the broad impact that age-related hearing loss has on the functioning of older adults and future health outcomes.^12^^–^^17^ A 2017 report from the Lancet Commission on Dementia concluded that hearing loss is the single largest modifiable risk factor for dementia.^18^ Importantly, the mechanisms underlying these observed associations may be modifiable with existing interventions, such as hearing aids.^19^ However, the prevalence of hearing aid use among adults who could potentially benefit remains low across the world, ranging from just a few percent in some countries to 20–30% in others.^5^^,^^20^^–^^23^ This can, in part, be attributed to the continuing high cost of hearing technologies universally, despite 30 years of exponential advances in computing and digital technologies and decreasing prices for nearly all consumer electronics.^17^^,^^24^

In this article we explore the financing and delivery systems for hearing aids for adults in several countries that are currently facing the challenges of an ageing society. We discuss areas where innovative policies and strategies could be employed to increase access to hearing aids.

## Hearing health care

We gathered data for this article through a wide search of the grey literature from August 2018 to April 2019, which included white papers, government reports, government websites, industry reports and regulatory association guidelines. The search was limited to English language sources. We used personal correspondence with academic and industry experts to address gaps in publicly available data.

We focus on eight middle- and high-income countries (Australia, Brazil, China, Germany, Japan, Netherlands, United Kingdom of Great Britain and Northern Ireland, and United States of America). We chose these countries to demonstrate variations across five factors that affect how hearing aids and related services are delivered: (i) availability of insurance coverage; (ii) training and scope of practice of hearing-care providers; (iii) pathways for obtaining hearing aids; (iv) where patients obtain their devices; and (v) how sales of hearing aids are regulated (Table 1).

**Table 1:** Hearing aid policies and purchasing pathways across eight middle- and high-income countries

Hearing aid policies and purchasing pathways	Australia^23^^,^^25^^–^^27^	Brazil^a^	China^28^^,^^29^^,b^	Germany^30^^,c^	Japan^31^^,d^	Netherlands^e^	United Kingdom^26^^,^^27^^,^^32^^–^^35^^,f^	United States^17^^,^^25^^,^^36^^,^^c^
**Types of providers^g^**	Audiologist; and hearing aid audiometrist	Audiologist; speech pathologist and hearing aid dispenser	Audiologist; hearing aid dispenser; nurse and hearing technician working in hospital; and ear, nose and throat specialist after audiology training	Hearing aid acoustician; master acoustician; and medical audiologist	Hearing aid technician and speech-language hearing therapist	Audiologist and hearing instrument specialist (equivalent to hearing aid dispenser)	Audiologist; Hearing aid dispenser; and clinical scientist (audiology)	Audiologist; hearing aid dispenser; physicians
**Training of providers**	Audiologist: generally a 2-year master degree with 1-year clinical internship, but there is no mandatory licensing of audiologists. Regulation is through the national professional body (Audiology Australia). Hearing aid audiometrist: minimum of diploma-level vocational training or undergraduate degree in audiometry	Audiologist: doctorate degree or university-level audiology specialization course regulated by the national professional body. Hearing aid dispensers: not regulated and can be anyone with some experience or informal training	In general, training through university, manufacturer, hospital, and in-house clinical training	Hearing aid acoustician: 3–4 years of training. Master acoustician: additional 1 year after hearing aid acoustician training. Medical audiologist: certified ear, nose and throat specialist physician with extra training in audiology	Hearing aid technician: must pass a technical test under the public-interest incorporated foundation (the Association for Technical Aids), to receive a private licence. Workshops are held for passing this test. Speech-language hearing therapist: must undergo specialized college training and pass a national qualification examination for licensing. They must gain practical experience at a certified hearing aid expert’s shop under the guidance of a hearing aid technician	Audiologist: regulated in a similar way as medical specialists, with 4 years of post-master degree education (master degree in physics or equivalent is required). Registered as medical physicists. Registration must be renewed every 5 years based on strict criteria. Hearing instrument specialist: post-secondary diploma, not regulated by law, but by quality control via professional bodies. Registration must be renewed every 5 years based on strict criteria.	Audiologist: master degree or bachelor degree. Hearing aid dispenser: diploma degree. Clinical scientist (audiology): scientist training programme or completion of the higher training scheme regulated by the British Academy of Audiology following an accredited master degree in audiological science	Audiologist: doctoral degree required. Licensed to practice by separate States, certification comes from centralized professional societies but is not needed to practise. Hearing aid dispenser: college diploma or high school diploma (according to State), with certain number of practical training hours required. Physician: Certified physician with extra training in audiology
**Scope of providers’ practice**	Audiologist: audiometric and special testing, diagnosis and dispensing of hearing aids. Hearing aid audiometrist: basic audiometric testing and dispensing of hearing aids	Audiologist or speech pathologist: audiometric and special testing, diagnosis and dispensing of hearing aids. Hearing aid dispenser: basic audiometric testing and dispensing of hearing aids	Health-care provider in hospital: audiometric and special testing, diagnosis and dispensing of hearing aids. Health-care provider at retail shop: simple audiometric testing and dispensing of hearing aids	Hearing aid acoustician: audiometric testing and dispensing of hearing aids. Master acoustician: audiometric and special testing, diagnosis, dispensing of hearing aids and supervision of hearing aid acousticians. Medical audiologist: audiometric and special testing, diagnosis, dispensing of hearing aids and medical and surgical treatment of hearing loss	Hearing aid technician: simple audiometric testing and dispensing of hearing aids. Speech-language hearing therapist: audiometric and special testing, diagnosis and dispensing of hearing aids	Audiologist: audiometric and special testing, diagnosis and dispensing of hearing aids. Hearing instrument specialist: simple audiometric testing and dispensing of hearing aids	Audiologist: audiometric and special testing, diagnosis and dispensing of hearing aids. Hearing aid dispenser: typically working in private practice to assess, fit and provide aftercare for hearing aids. Clinical scientist: (similar to audiologist) audiometric and special testing, diagnosis, dispensing of hearing aids	Audiologist: audiometric and special testing, diagnosis and dispensing of hearing aids. Hearing aid dispenser: basic audiometric testing and dispensing of hearing aids. Physician: audiometric and special testing, diagnosis, dispensing of hearing aids and medical and surgical treatment of hearing loss
**Insurance coverage for hearing aids**	Public insurance: certain groups have partial to full coverage of costs. Pension concession cardholders and war veterans are covered through the Office of Hearing Services Voucher Scheme. Children aged 26 years or younger and at-risk patients with complex disease and Aboriginal and Torres Strait Islander peoples are covered through the Community Service Obligation scheme. Older patients with disabilities are covered through the National Disability Insurance Scheme. Majority of adults over 26 years of age pay out-of-pocket	Public insurance: full coverage of costs under the unified health system (*Sistema Unico de Saude*). Majority of patients pay out-of-pocket	Public insurance: each province has a different policy on reimbursement. Most patients have private insurance coverage or pay out-of-pocket	Public insurance: partial to full coverage for those earning less than about 60 000 euros a year. Those earning more than 60 000 euros a year can opt-in to the public insurance coverage scheme with a fee. Minority of patients pay out-of-pocket for premium hearing aids only	Public insurance: limited partial coverage of costs for children, according to State, and to patients with disabling hearing loss for whom a physically disabled certificate is issued (under the Physically Disabled Welfare Act). Most patients pay out-of-pocket	Public insurance: full coverage of costs with a 25% statutory contribution by the patient. Private insurance: additional coverage of costs available through supplemental insurance	Public insurance: full coverage of costs through the National Health Service A minority of patients pay out-of-pocket for premium hearing aids only	Public insurance: partial to full coverage of costs under Medicaid, Medicare Advantage (part C) and Veterans Affairs. Private insurance: largely employee-sponsored and through other third-party insurers. Most patients pay out-of-pocket
**Hearing aids covered by insurance**	Voucher scheme covers mostly basic models of hearing aids. Community Service Obligation scheme covers a greater range of hearing aids available, due to the complexity of disease. Disability Scheme covers all major types of hearing devices. Patients can pay extra to purchase premium products	Insurance covers basic and premium models of hearing aids to different percentages. Varies according to State. Patients who pay out-of-pocket can purchase any device	Insurance covers the generation of technology older than the latest hearing aids. Patients who pay out-of-pocket can purchase any device	Insurance covers basic models of hearing aids. Patients can generally pay extra to purchase premium products. Patients who pay out-of-pocket can purchase any device	Public subsidy for select groups covers basic models of hearing aids, but no insurance coverage is available	Insurance covers purchase of a new or replacement set of hearing aids every 5 years. Batteries are paid for by patient	Insurance covers basic models of hearing aids. Patients who pay out-of-pocket can purchase any device	Insurance covers basic models of hearing aids. Patients can generally pay extra to purchase premium products (except for Veteran Affairs and Medicaid). Patients who pay out-of-pocket can purchase any device
**Availability of hearing aids**	Audiology departments at major hospitals; Australian Hearing Services clinics; audiologist offices in private practice; commercial hearing aid providers	Audiologist offices; hospitals; retail clinics or hearing aid shops	Hospitals; retail shops; online shops pharmacies; rehabilitation centres for deaf children; disabled assistive apparatus centres	Independent providers; retail clinics or shop chains	When hearing aid technicians are employed, hearing aids are available at the following: department stores; optical shops; home appliance retailers; online shops	Contracted care providers: retail clinics combined with optical shops that have an agreement with the public insurance system. Non-contracted care providers: retail clinics that are not contracted, but still must meet guideline criteria for selling. Hearing aid dispensers: contracted , but still must meet guideline criteria for selling	National Health Service hospitals; contracted private clinics (through any qualified provider scheme); private clinics (retail); and independent clinics	Audiologist offices; hearing aid offices; physician offices; hospitals; retail clinics; and online shops
**Regulation of hearing aids approved for sale**	Therapeutic Goods Administration	*Agencia Nacional de Vigilancia Sanitaria*	China Food and Drug Administration	German Hearing Aid Institute. Statutory health insurance body (*gesetzliche Kranken-versicherung*)	Hearing aids are registered by the Medical Health Board	European Union: Medical Device Directive	The Medicines and Healthcare Products Regulatory Agency working with the European Union: Medical Device Directive, which implements the Medical Devices Regulation	United Sates Food and Drug Administration
**Hearing aid purchasing pathways^h^**	Patients must have medical clearance from a physician before referral to an audiologist or hearing aid audiometrist	All patients must have medical clearance by a physician to before being referred to an audiologist or a hearing aid dispenser. Patients purchase devices from an audiologist or hearing aid dispenser	In hospital, patients must have medical clearance from an audiologist or physician. In private hearing aid shops, no clearance by a professional is needed	Patients with insurance must obtain a prescription for the first hearing aid through an ear, nose and throat specialist. Patients with no insurance can purchase devices directly from an acoustician, but this is uncommon.	Patients with hearing loss can freely purchase hearing aids at certified hearing aid expert shops where hearing aid technicians and speech-language-hearing therapists work	Patients can go directly to an audiologist or hearing aid dispenser to purchase a device. Dispensers must refer the patient to an ear, nose and throat specialist or audiological centre to be medically assessed if there are medical concerns on testing (severe hearing loss, asymmetric hearing loss, air–bone gap, poor speech recognition, young patients)	Patients must be referred by a general practitioner or ear, nose and throat specialist to access National Health Service hearing aid services (either in hospital or through any qualified provider services). Patients can purchase devices from an audiologist or hearing aid dispenser. Patients can self-refer to independent or retail providers and purchase hearing aids	All patients require medical clearance from an audiologist, general practitioner, or ear, nose and throat specialist to obtain a hearing aid, or must sign a medical waiver. Patients can purchase devices from an audiologist, hearing aid dispenser or other professional licensed to dispense hearing aids. By 2020, the Over-the-Counter Act passed in 2017 will create a Food and Drug Administration regulatory classification for hearing aids intended for mild and mild-to-moderate hearing loss that are directly available over the counter to adults
**Cost per hearing aid (without insurance)**	Typical price range: US$ 1400–2800	Typical price range: US$ 780–3900	Economy price range: US$ 230–670. Mid–high price range: US$ 1200–1770. High price range: US$ 1500–3000. Top price range: US$ 4 500–6000	Typical price range: US$ 900–4250	Behind-the-ear or receiver-in the-canal hearing aids typical price range: US$ 453–4530	Typical price range: US$ 1000–3000	Typical price range: US$ 650–4500	Typical price range: US$ 500–3000. Economy price range: US$ 300–500 (through mail order and retail stores)

US$: United States dollars (exchange rate at 1 May 2018).

^a^ Goffi V, University of Sao Paulo, Brazil, personal communication, 2018; Lopes A, Hearing Solutions, Brazil, personal communication, 2018.

^b^ Fu X, Beijing Tong Ren Hospital, China, personal communication, 2018.

^c^ Baldwin D, Unitron, Switzerland, personal communication, 2018.

^d^ Hayashi K, Keio University, Japan, personal communication, 2018.

^e^ Hoetink A, University Medical Center Utrecht, Netherlands, personal communication, 2018; Snik A, Radboud University Medical Centre, Netherlands, personal communication, 2018.

^f^ Bamiou D, University College London, United Kingdom, personal communication, 2018; Tutton M, University College London, United Kingdom, personal communication, 2018.

^g^ Audiologists are highly-trained clinicians who provide a range of hearing-care services, basic and complex diagnostic hearing and balance testing, hearing-care counselling and fitting and adjustment of hearing devices. They are typically not physicians and cannot administer medical or surgical treatment. Hearing aid dispensers are individuals who typically focus on fitting and dispensing hearing aids to patients. They also frequently perform basic diagnostic hearing testing and hearing-care counselling.

^h^ Medical clearance refers to a patient who has undergone a medical evaluation by a physician. The term referral means that a patient is being sent to another health-care professional to receive appropriate services. Patients receive medical clearance from a physician once there are no medically-treatable conditions identified. Patients are referred to (given an appointment to see) an audiologist or hearing aid audiometrist for purchasing a hearing aid. Medical waivers are forms that patients must sign to indicate that they understand that they are bypassing a recommended medical evaluation before purchasing a hearing aid.

### Insurance coverage

The costs associated with purchase of a hearing aid may be covered by public insurance, private insurance or out-of-pocket payments by the patient at the point of purchase (or combinations of these).

Public insurance for hearing aid purchases and services varies widely across countries by the population covered, device options and generosity of benefits. In the United Kingdom, all patients have access to public coverage of the costs of hearing aids whereas Australia and the United States restrict social insurance coverage of hearing aids to older adults who meet qualifying criteria, such as low income.^17^^,^^25^^,^^32^ Some European countries, such as the Netherlands and the United Kingdom, provide extensive financial coverage, reimbursing nearly 100% of costs incurred by the patient.^37^^,^^38^ In countries that have insurance coverage for hearing aids, patients may generally also pay out-of-pocket to gain access to premium devices, such as in Australia, Brazil, Germany, the United Kingdom, and the United States (Hoetink A, University Medical Center Utrecht, Netherlands, personal communication, 2018; Snik A, Radboud University Medical Centre, Netherlands, personal communication, 2018; Baldwin D, Unitron, personal communication, 2018; Szklo M, Johns Hopkins University, United States, personal communication, 2018).^25^^,^^26^^,^^37^^–^^41^ Countries such as China and Japan provide little to no central public insurance coverage (Hayashi K, Keio University, Japan, personal communication, 2018; Fu X, Beijing Tong Ren Hospital, China, personal communication, 2018).

Private insurance and out-of-pocket payments are the main means of financial access to hearing aids for a high proportion of patients worldwide (Hayashi K, 2018; Fu X, 2018; Szklo M, 2018).^26^^,^^39^^–^^41^ For example, the majority of adults living in the United States bear the total cost of hearing aids because of limited scope and coverage of employer-sponsored or third-party insurance plans.^17^ Patients in China rely on private insurance and out-of-pocket payments for hearing care, while in Japan most individuals pay out-of-pocket for hearing devices and services (Hayashi K, 2018; Fu X, 2018).

### Hearing aid providers

The two major categories of hearing aid providers, according to the scope of care required, are audiologists and hearing aid dispensers (also known as hearing aid audiometrists in Australia), with contributions from various other professionals (Hoetink A; 2018; Snik A, 2018; Baldwin D, 2018; Hayashi K, 2018; Fu X, 2018).^17^^,^^21^^,^^25^^,^^27^^,^^30^^–^^33^^,^^37^^,^^40^^–^^43^ The training required for becoming a provider is highly variable across countries and even within the same country, ranging from informal training for hearing aid dispensers to a doctorate degree for audiologists. Licensing is often decentralized to the state or province level.

### Purchasing pathways

The purchasing pathway refers to the steps through which a patient requiring a hearing aid must proceed, starting from the identification of a deficit in hearing to the point they acquire a hearing aid. In most countries providing public insurance, patients must receive a diagnostic hearing test and consult an audiologist, a general physician or an ear, nose and throat specialist before being eligible to purchase a hearing aid (Baldwin D, 2018; Goffi V, University of Sao Paulo, Brazil, personal communication, 2018; Lopes A, Hearing Solutions, Brazil, personal communication, 2018).^17^^,^^25^^,^^37^^,^^44^ This procedure is to rule out medically-treatable causes of hearing loss. The United States requires all patients, regardless of public insurance coverage status, to consult one of the above professionals before purchasing a hearing aid.^17^^,^^25^^,^^32^ However, the United States passed a law in 2017 which will soon allow some patients to purchase hearing aids directly over the counter, bypassing the need to first seek a professional opinion.^45^

Other countries are less stringent on the requirement of visiting a hearing-care professional before purchasing hearing aids. In the Netherlands, patients can proceed directly to a hearing-aid dispenser for a hearing assessment and purchase of devices (Hoetink A, 2018; Snik A, 2018). In Australia, Germany and the United Kingdom, patients who are not receiving public insurance coverage can directly purchase hearing aids from hearing-aid dispensers (Baldwin D, 2018; Bamiou D, University College London, United Kingdom, personal communication, 2018; Tutton M, University College London, United Kingdom, personal communication, 2018). China also allows patients who visit hearing-aid shops or clinics outside of hospitals to purchase their devices directly (Fu X, 2018). In Japan, where insurance is non-existent, patients are free to purchase hearing aids at their own discretion (Hayashi K, 2018).

### Purchase of hearing aids

One pathway to purchasing devices is at the hospital or office of the hearing-care provider where patients undergo their medical consultations and diagnostic testing. However, patients are increasingly using conveniently located and diversified retail clinics, which range from small private vendors to large chain stores (Hayashi K, 2018).^17^^,^^25^ Public insurance programmes in Australia, the Netherlands and the United Kingdom contract-out hearing aid dispensing services to accredited retail clinics or qualified providers (Hoetink A, 2018; Snik A, 2018; Tutton M, 2018).^25^^,^^37^ Large retail clinics in department stores, such as those in the United States, attract customers due to their ability to decrease costs from economies of scale, improve accessibility and shorten waiting times.^17^ In Japan, joint hearing and optical services provide a large proportion of hearing-aid services to the public, a model which is emerging in Australia and the United Kingdom (Hayashi K, 2018). Lastly, online sales have been slowly growing in popularity globally, with regions such as Japan realizing considerable sales through this method (Hayashi K, 2018).

### Regulation of sales

In the eight countries examined, hearing aids are classified as medical devices within regulatory bodies that dictate strict criteria that must be met before sale in the market. Current regulations around the sale of hearing aids can limit access for some patients due to the requirement for medical clearance by a designated health professional before purchase. However, it is important to note that while hearing aids are regulated, devices classified as personal sound amplification products are generally not (Box 1).^28^^,^^34^

Box 1 **Definitions of hearing aids and personal sound amplification products in the United States of America**

Hearing aids are regulated by the United States Food and Drug Administration (FDA) as class I or class II medical devices. They are defined as “any wearable instrument or device designed for, offered for the purpose of, or represented as aiding persons with or compensating for, impaired hearing” (21 CFR 801.420).^44^ As regulated devices, hearing aids are distributed through licensed individuals and they are able to make claims, which are regulated by the Federal Trade Commission, about treating hearing loss. Hearing aids are suitable for any degree of hearing loss.

Personal sound amplification products are over-the-counter electronic devices that are not regulated by the FDA. The FDA has issued guidance that these devices are “intended to amplify environmental sound for non-hearing impaired users… They are not intended to compensate for hearing impairment or to address listening situations that are typically associated with and indicative of hearing loss.”^44^ As personal sound amplification devices are an unregulated consumer product intended for those without hearing loss, distributors are restricted in their advertising and cannot make claims about addressing hearing loss.

## Opportunities for innovation

While funding and delivery of hearing care vary substantially from country to country, there are several common areas where innovations could potentially lead to easier access and increased uptake of hearing aids for adults with hearing loss.

### Regulatory innovations 

Current regulations in most countries stipulate that hearing aids can only be sold or dispensed through a licensed provider to maximize the safety and efficacy of devices. For example, with older analogue hearing aids, calibrating and properly fitting a hearing aid could involve crafting a custom ear-mould to minimize audio feedback and manually adjusting trim potentiometers on the hearing aid to set appropriate levels of gain.

Over the past 10–15 years, however, innovations have been made in digital circuitry and technologies that allow hearing aids to be self-fitted and set to appropriate levels of audio output to avoid hearing damage. Research demonstrates that such hearing aids are both safe and effective for adults with mild-to-moderate hearing loss, but regulatory models in most countries preclude such devices from being sold directly to patients.^29^^,^^46^^,^^47^ In other countries with unregulated hearing-aid markets, such as in Japan, these devices can potentially be sold, although patients have no way of distinguishing which devices are safe and effective.

Recent initiatives in the United States from the National Academies of Science, Engineering and Medicine and the White House President’s Council of Advisors on Science and Technology, suggest a new regulatory model to allow for greater access to hearing aids.^17^^,^^48^ Recommendations from these bodies led to the passage of federal legislation in 2017 that will result in a regulated market for over-the-counter hearing aids in the country.^45^ A key feature of this legislation is that the Food and Drug Administration will define explicit performance and labelling criteria by 2020 to ensure that hearing aids purchased over the counter are both safe and effective. Both the National Academies and President’s Council anticipate that the implementation of a transparent market for over-the-counter hearing aid sales will allow for greater competition by lowering market entry barriers for new companies, thereby leading to increased technological innovation, reduced costs and broader access to hearing aids.^17^^,^^48^

### Technological innovations

A crucial building block for the feasibility of over-the-counter sales regulations in any country is reliable technology. Advances in digital amplification technologies over the past few decades have led to the emergence of products that could meet the needs of a direct-to-consumer amplification device. In the United States, this resulted in a class of unregulated amplification devices called personal sound amplification products, some of which have similar traits as traditional hearing aids (Box 1).^28^^,^^43^ These products follow a trend across health care whereby mass consumer companies are now producing devices that have the same capabilities as medical devices.^49^

Many of these devices include now-standard digital hearing aid technologies such as noise-reduction algorithms, directionality and linked remote microphones to tackle hearing in difficult situations. Some key innovations too are empowering individuals with hearing loss to benefit better from amplification products. Feedback suppression and advances in durable plastics allow for non-custom ear tips. Additionally, integration with smartphone applications allows for self-adjustment of hearing aids by the user, with the phone delivering a hearing test and the device performing in situ customization to prescriptive targets. Moreover, changes in battery technology allow for rechargeable units that are easier to manipulate.^28^

While previous research revealed that low-cost, inappropriate technology produces poor electroacoustic results, recent studies have demonstrated that some direct-to-consumer devices are technologically similar to traditional hearing aids.^46^^,^^48^^,^^50^ Similar results were found in a more difficult noise environment, comparing personal sound amplification products adjusted by the user and traditional hearing aids adjusted by a professional.^47^ Nevertheless, many current direct-to-consumer devices have been found to produce poor electroacoustic measures.^35^^,^^50^ Regulation of direct-to-consumer amplification devices via strict performance and labelling criteria would help eliminate these inferior devices in the market and allow for robust, consumer-oriented selection of hearing devices. While there are potential safety concerns with patients having direct access to over-the-counter sales of hearing aids, including missed cases of medically or surgically treatable hearing loss, the benefits of allowing adults broader access to hearing aids far outweigh the potential risks.^17^^,^^48^

### Reimbursement innovations

For many health systems, the availability of over-the-counter or direct-to-consumer hearing aids has the potential to disrupt the delivery model for hearing aids and hearing services. Systems that pay for hearing-care services independently of devices may be able to adapt more readily than systems (such as in the United States) that operate on a predominantly bundled reimbursement model where the reimbursement of hearing-care services is routinely coupled with the cost of the device. As these systems evolve with the changing distribution landscape, two important factors require consideration.

First, ensuring that patients can continue to access hearing-care services independent of the hearing technology used is of importance. Hearing-care services, provided by audiologists or other hearing-care providers, are a fundamental component to successful hearing aid use. These tasks include orienting individuals to the device, providing fitting and customization specific to the patient’s hearing needs, and providing supportive communication strategies and techniques.^51^

A second important factor is the reimbursement of new hearing-aid devices in an expanded marketplace. Ensuring that the availability of over-the-counter hearing aids does not lead to the elimination of insurance coverage is important. Given that the most common pathway to purchase hearing aids is through hearing-care providers, policy-makers must consider how to adapt to a wider array of products available for over-the-counter purchase. The extent of change required depends greatly on how the system of reimbursement currently operates. For example, in the United Kingdom, where publicly-funded insurance coverage is widespread and choice of devices covered by insurance is limited, adapting to an expanded hearing aid marketplace will require substantial change.^37^ This adaption process will require strong regulatory guidance regarding the over-the-counter hearing care products that qualify for reimbursement, as well as a mechanism by which patients can submit a claim for reimbursement of that purchase.

### Workforce innovations

In addition to the potential impact of a shifting hearing aid market on the ability for patients to access devices, the persistently low uptake of hearing aids worldwide indicates that the health behaviour of patients plays a major role in hearing care.^5^^,^^52^^,^^53^ Older adults are often on fixed incomes, with physical and mental limitations and multiple chronic medical conditions that create competing priorities. For this group, factors that influence their ability and willingness to access clinic-based care include financial resources, provider availability, service location, patient mobility and their personal health beliefs and acceptance of technology.^54^ To ensure that services reach those who need them, health-care delivery models that address effective technology use, limited insurance coverage, provider availability and sociocultural barriers are needed, namely through community-delivered models of care.

Community-delivered hearing care broadly encompasses care delivered beyond traditional clinic-based services and incorporates task-sharing through paraprofessionals, such as community health workers, health aides or peer mentors. The community health worker model has been widely adapted for use in diverse settings in low- and middle-income countries.^36^^,^^55^^,^^56^ Community health workers often share the same language, cultural background and lived experiences as their patients and are uniquely positioned to address hearing loss as a complex health behaviour.^57^

Growing efforts around the world are using community-delivered hearing care to older adults. In the United States, two programmes in low-resource settings have been developed. *Oyendo Bien* consists of group aural rehabilitation sessions for older adults with hearing loss who live along the United States–Mexico border, led by *promotoras* (lay community workers with basic hearing health-care training).^58^ Similarly, the Hearing Equality through Accessible Research and Solutions programme focuses on low-income, urban, older adults and extends the services delivered by a paraprofessional to include fitting and orientation to low-cost, high-quality over-the-counter amplification devices.^59^ In India, through a public–private partnership, the Shruti project has trained a cohort of community health workers who perform hearing screening and fit adults with low-cost hearing aids.^30^^,^^60^ Finally, World Wide Hearing, a nongovernmental organization based in Canada, has pioneered the delivery of technician-provided hearing care for children in low-resource settings and has now begun to expand this model to adults.^61^ These new models of community-delivered hearing care provide initial evidence that trained paraprofessionals can provide safe, high-quality hearing care and directly address barriers to traditional clinic-based care.

## Conclusion

A country’s hearing health-care system plays an important role in how well society can address the global health priority of hearing loss. A steadily ageing global population and the generally low prevalence of hearing aid use makes it imperative that innovations in policy and practice are implemented to meet the evolving challenges that hearing loss will present in the near future. The wide spectrum of delivery models, hearing-care providers and reimbursement models that we have highlighted in this article provides an overview of the current state of hearing health care across higher-income countries and the possibilities for advancement. Innovations in hearing aid regulation, technology, reimbursement schemes and workforce models provide tangible actions for WHO Member States to consider in addressing the growing global burden of hearing loss and making hearing health a political priority.
